# Transient Receptor Potential Ankyrin 1 Channel Localized to Non-Neuronal Airway Cells Promotes Non-Neurogenic Inflammation

**DOI:** 10.1371/journal.pone.0042454

**Published:** 2012-08-14

**Authors:** Romina Nassini, Pamela Pedretti, Nadia Moretto, Camilla Fusi, Chiara Carnini, Fabrizio Facchinetti, Arturo Roberto Viscomi, Anna Rita Pisano, Susan Stokesberry, Charlott Brunmark, Naila Svitacheva, Lorcan McGarvey, Riccardo Patacchini, Anders B. Damholt, Pierangelo Geppetti, Serena Materazzi

**Affiliations:** 1 Department of Preclinical and Clinical Pharmacology, University of Florence, Florence, Italy; 2 Pharmacology Department, Chiesi Farmaceutici SpA, Parma, Italy; 3 Department of Biochemistry and Molecular Biology, University of Parma, Italy; 4 Centre for Infection and Immunity, Queen's University Belfast, Belfast, United Kingdom; 5 AstraZeneca Research & Development Innovative Medicines Respiratory & Inflammation, Mölndal, Sweden; 6 Truly Translational Sweden AB, Lund, Sweden; 7 Disease Pharmacology LEO Pharma A/S, Ballerup, Denmark; 8 Department of Biology, University of Copenhagen, Copenhagen, Denmark; 9 Headache Center, University of Florence, Florence, Italy; Cinvestav-IPN, Mexico

## Abstract

**Background:**

The transient receptor potential ankyrin 1 (TRPA1) channel, localized to airway sensory nerves, has been proposed to mediate airway inflammation evoked by allergen and cigarette smoke (CS) in rodents, *via* a neurogenic mechanism. However the limited clinical evidence for the role of neurogenic inflammation in asthma or chronic obstructive pulmonary disease raises an alternative possibility that airway inflammation is promoted by non-neuronal TRPA1.

**Methodology/Principal Findings:**

By using Real-Time PCR and calcium imaging, we found that cultured human airway cells, including fibroblasts, epithelial and smooth muscle cells express functional TRPA1 channels. By using immunohistochemistry, TRPA1 staining was observed in airway epithelial and smooth muscle cells in sections taken from human airways and lung, and from airways and lung of wild-type, but not TRPA1-deficient mice. In cultured human airway epithelial and smooth muscle cells and fibroblasts, acrolein and CS extract evoked IL-8 release, a response selectively reduced by TRPA1 antagonists. Capsaicin, agonist of the transient receptor potential vanilloid 1 (TRPV1), a channel co-expressed with TRPA1 by airway sensory nerves, and acrolein or CS (TRPA1 agonists), or the neuropeptide substance P (SP), which is released from sensory nerve terminals by capsaicin, acrolein or CS), produced neurogenic inflammation in mouse airways. However, only acrolein and CS, but not capsaicin or SP, released the keratinocyte chemoattractant (CXCL-1/KC, IL-8 analogue) in bronchoalveolar lavage (BAL) fluid of wild-type mice. This effect of TRPA1 agonists was attenuated by TRPA1 antagonism or in TRPA1-deficient mice, but not by pharmacological ablation of sensory nerves.

**Conclusions:**

Our results demonstrate that, although either TRPV1 or TRPA1 activation causes airway neurogenic inflammation, solely TRPA1 activation orchestrates an additional inflammatory response which is not neurogenic. This finding suggests that non-neuronal TRPA1 in the airways is functional and potentially capable of contributing to inflammatory airway diseases.

## Introduction

A subset of primary sensory neurons expresses and releases the neuropeptides calcitonin gene-related peptide (CGRP) and tachykinins, substance P (SP) and neurokinin A (NKA), producing a series of inflammatory responses, collectively referred to as neurogenic inflammation [1]. Airway neurogenic inflammation, mainly mediated by SP and NKA, *via* the activation of NK1 and NK2 receptors, encompasses a series of transient phenomena, including plasma protein extravasation and neutrophil adhesion to the vascular endothelium, which have been implicated in the mechanisms of airway inflammatory diseases, including asthma and chronic obstructive pulmonary diseases (COPD) [1], [2]. In experimental animals, SP/NKA contribute to the early inflammatory response evoked by common aggravants of asthma and COPD, such as allergens [3] and cigarette smoke [4]. However, despite decades of intense research, there is little [5] or no evidence that neuropeptide activation of NK1/NK2 receptors is responsible for the typical features of asthma or COPD, including cytokine release, chronic inflammatory cell infiltration, and airway hyperresponsiveness.

Peptidergic sensory neurons express the transient receptor potential vanilloid 1, (TRPV1) [6] and ankyrin 1 (TRPA1) channels [7], [8], [9], [10]. TRPV1 stimulants, such as capsaicin, low extracellular pH, and certain lipid derivatives [6], [11], [12], [13]), or TRPA1 stimulants, such as several exogenous pollutants with toxic liability and a host of endogenous by-products of oxidative and nitrative stress [14], [15], [16], [17], [18], [19], release sensory neuropeptides and produce airway neurogenic inflammation [20], [21]. Recently, it has been shown that activation of TRPA1, but not TRPV1, modulates airway inflammatory response in murine models of allergic asthma, reactive airways dysfunction syndrome (RADS) and COPD, induced by cigarette smoke [20], reactive acetaminophen metabolite [21] or allergen [22]. Such observations have advanced the hypothesis that airway sensory nerves *via* a neurogenic inflammatory mechanism mediate these clinical phenomena. However, as clinical trials in the last 20 years with NK1/NK2 receptor antagonists have shown little efficacy in asthma [23], [24], and, with no reported therapeutic role so far reported by the use of NK1/NK2 receptor antagonists in COPD, it seems unlikely that SP/NKA released from sensory nerves are the sole and the major contributors of TRPA1-mediated inflammation in the airways.

The apparent contradiction that activation of both TRPV1 and TRPA1 causes neurogenic airway inflammation, but only TRPA1 agonists produce hallmark features of airway inflammation in models of asthma and COPD [20], [22], [25], could be explained if TRPA1 channels were expressed not only by sensory nerves, but also by non-neuronal cells of the airways, from which they orchestrate neurogenic-independent inflammatory responses. Recent identification of functional TRPA1 channel in enterochromaffin cells of the gastrointestinal tract [26] vascular endothelial cells [27], and keratinocytes [28] support the proposal that TRPA1 agonists may act on channels expressed by non-neuronal cells to promote key inflammatory responses. Here we present evidence that non-neuronal cells in human and murine airways express TRPA1, where it promotes a non-neurogenic inflammatory response, which may contribute to asthma and COPD.

## Results

### TRPA1 expression in non-neuronal cells of the human and mouse respiratory tract

**Figure 1:**
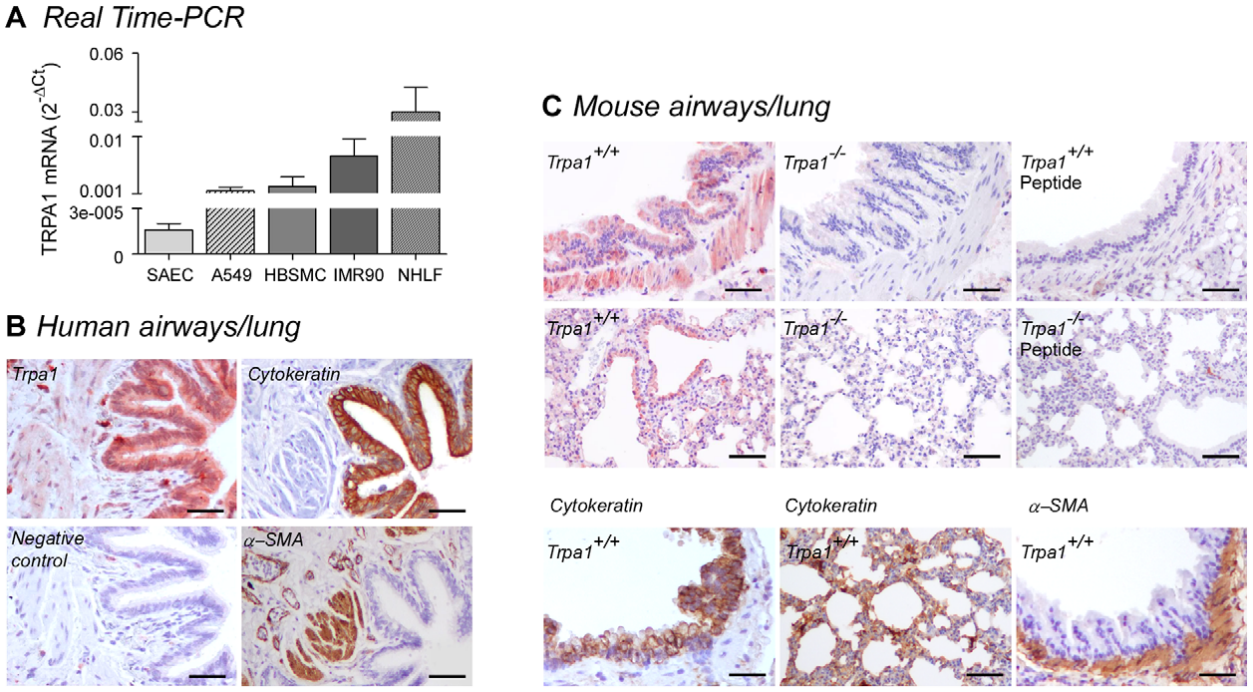
TRPA1 channel expression in non-neuronal cells of the human and mouse respiratory tract. (**A**) Total RNAs were extracted from primary small airways epithelial cells (SAEC), human type II alveolar epithelial cells (A549), human primary smooth muscle cells (HBSMC), human embryonic lung fibroblasts (IMR90) and primary normal human lung fibroblasts (NHLF) and relative TRPA1 mRNA amounts were measured by Taqman Real-Time PCR assay. Results are normalized to the reference gene, β-actin. Each column represents mean ± SEM of n>2 independent experiments. Immunohistochemical analysis of TRPA1 expression in samples taken from human (**B**) or *Trpa1^+/+^* and *Trpa1^−/−^* mouse airways and lung (**C**). Representative images of TRPA1 immunostaining show intense staining in epithelial and smooth muscle cells in human tissue. No staining is detected in human samples incubated with the normal serum peptide (Negative control). (**C**) Incubation with TRPA1 antibody shows a strong staining in epithelial and smooth muscle cells in tissues taken from *Trpa1^+/+^* mice, but not in those from *Trpa1^−/−^* mice. Preadsorption of the TRPA1 antibody with the peptide used for immunization abolished staining (Peptide). Staining for cytokeratin and α-smooth muscle actin (α-SMA) overlaps with the TRPA1 staining in the bronchial epithelium and smooth muscle layer in serial section of human and mice airways/lung tissues (**B** and **C**). Scale bar 100 µm.

To assess the expression of TRPA1 in different types of human airway/pulmonary cells, (either immortalized cell lines or primary cultures), we performed Real-Time PCR on total mRNA isolated from human alveolar type II epithelium-like adherent cell line (A549), human small airway epithelial cells (SAEC), human embryonic lung fibroblasts (IMR90), normal human lung fibroblasts (NHLF), and human bronchial smooth muscle cells (HBSMC). Using specific TaqMan primers and probe sets, we found detectable levels of human TRPA1 transcript in all the examined cells (Figure 1A). To investigate the expression of TRPA1 protein in non-neuronal cells of human airways and lung we performed an immunohistochemical evaluation with a specific human TRPA1 antibody [29]. Replacing the primary antibody with normal serum abolished the intense staining, present in epithelial and smooth muscle cells (Figure 1B). TRPA1 staining was also identified in non-neuronal cells of airway/lung tissue taken from wild-type (*Trpa1^+/+^*), but not in TRPA1-deficient (*Trpa1^−/−^*) mice (Figure 1C), by using a TRPA1 antibody that recognizes the middle region of the protein. In particular, intense staining was detected in the bronchial epithelium and smooth muscle layer. Staining was completely absent when the TRPA1 antibody was pre-adsorbed with the immunizing peptide (Figure 1C), confirming specificity. Absence of staining in trigeminal ganglion (an area enriched with TRPA1 expressing neurons) from *Trpa1^−/−^* mice, compared with the marked protein expression in ganglia taken from *Trpa1^+/+^* mice (Figure S1A), conclusively supports the specificity of the antiserum, and, accordingly, the immunohistochemical findings obtained in the airway. In slices of human and mouse airway/lung, antibodies for specific markers for epithelial cells (cytokeratin) or α-smooth muscle cells (α-smooth muscle actin) stained, as predicted, bronchial/lung epithelial and smooth muscle layers, respectively. A similar pattern of staining was found in both human and mouse serial sections with the antibodies for the human or mouse TRPA1, respectively (Figure 1B and C). By using immunofluorescence, staining for cytokeratin and TRPA1 merged in a large proportion of epithelial cells and staining for α-smooth muscle actin and TRPA1 merged in smooth muscle cells both in human and mouse lung tissues (Figure S1B and C). In additional experiments, using a TRPV1 antiserum which recognizes both human and mouse protein [30], we found that TRPV1 staining was absent in epithelial and smooth muscle cell layers in both human and mouse airways (Figure S2A and B). Mouse trigeminal ganglia have been used as control (Figure S2C).

### Airway/lung cells express a functional TRPA1 channel

**Figure 2:**
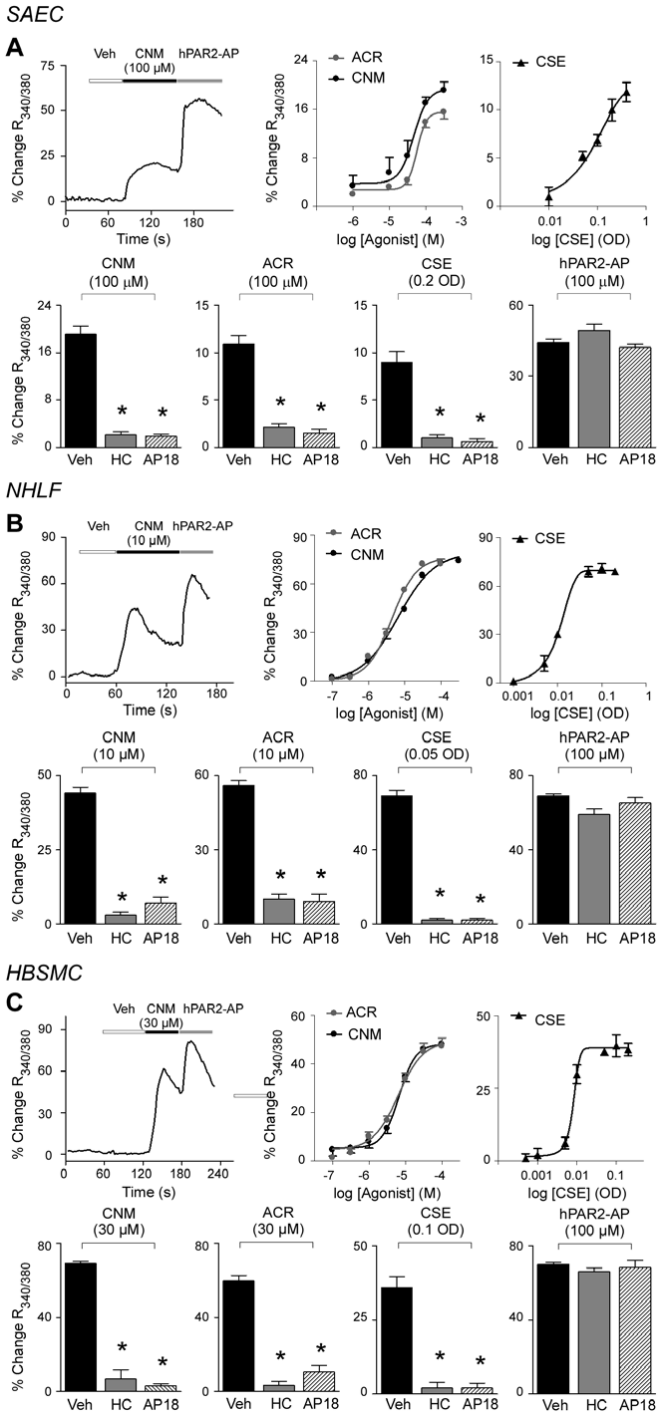
Functional TRPA1 is expressed in human airway/lung cells. Intracellular calcium response was used to assess agonist-induced TRPA1 activation in small airways epithelial cells (SAEC) (**A**), normal human lung fibroblasts (NHLF) (**B**) and bronchial smooth muscle cells (HBSMC) (**C**). Typical traces and pooled data of the concentration-dependent calcium response evoked by the selective TRPA1 agonists, cinnamaldehyde (CNM, typical traces and black circles) and acrolein (ACR, grey circles), in all different cell types in primary culture. Similarly to CNM and ACR, cigarette smoke extract (CSE, black triangles) produces in all the different types of cells a concentration-dependent calcium response. Responses to CNM, ACR and CSE are prevented by selective TRPA1 antagonists, HC-030031 (HC, 10 µM) and AP18 (10 µM). The activating peptide (SLIGKV-NH_2_) of the PAR-2 receptor (PAR-2 AP, 100 µM) elicits a calcium response that is not modified by TRPA1 antagonists. Veh is a combination of vehicles of HC and AP18. Values represent mean ± SEM of n>25 cells. ^*^ *P*<0.05 *vs.* Veh.

TRP channels are generally non-selective cation channels, and TRPA1 stimulation results in a massive influx of calcium into the stimulated cells [9]. Thus, to verify if mRNA and the resulting protein expressed by the different types of human airway cells produces and represents a functional channel, respectively, we exposed fibroblasts (IMR90 and NHLF), epithelial (A549 and SAEC), and smooth muscle (HBSMC) cells to TRPA1 agonists, including cinnamaldehyde [31], acrolein [14], and cigarette smoke aqueous extract (CSE). CSE contains more than 4,000 chemical compounds [32], and some of them have been previously identified as TRPA1 activators [20], [33], [34]. Administration of all the three TRPA1 agonists produced concentration-dependent elevation in intracellular calcium of slightly different intensity in all tested cells (Table 1). It should be underlined that the intensity of the response to TRPA1 agonists may be affected by changes in expression occurring in cell lines or even cultured cells. Thus, if the qualitative significance of the results is obvious, their quantitative value as predictor of the in vivo response to TRPA1 agonists remains to be determined. The TRPA1 selective antagonists, HC-030031 [35] or AP18 [36], invariably prevented the effects of submaximal agonist concentrations (Figure 2A–C and Figure S3A–C). HC-030031 or AP18 did not affect the response to the activating peptide of the PAR-2 receptor, thus indicating selectivity of the pharmacological intervention. The observation that calcium response to CSE was completely abated by TRPA1 antagonists indicates that calcium response caused by CSE is entirely mediated by TRPA1 stimulation. The selective and potent TRPV1 agonist, capsaicin (1 µM), failed to produce any visible calcium response in SAEC, A549, NHLF, IMR90, and HBSMC cells (data not shown).

**Table 1:** Potency (EC_50_ and CI) of the various TRPA1 agonists in different non-neuronal cell types of the human respiratory tract.

	*Cinnamaldehyde EC_50_ (CI) µM*	*Acrolein EC_50_ (CI) µM*	*Cigarette Smoke Extract EC_50_ (CI) OD*
**A549**	10.9 (6.4–18.6)	7.3 (4.7–11.4)	0.006 (0.001–0.05)
**SAEC**	46.3 (25.9–82.9)	56.9 (35.1–92.4)	0.026 (0.01–0.05)
**IMR90**	2.7 (1.3–5.3)	2.9 (1.2–7.6)	0.05 (0.02–0.07)
**NHLF**	6.6 (4.9–8.8)	4.4 (3.4–5.7)	0.01 (0.001–0.02)
**HBSMC**	6.7 (5.57–8.1)	6.3 (4.0–10.0)	0.008 (0.006–0.010)

### In vivo exposure to acrolein or cigarette smoke (CS) induces KC release via a non-neuronal mechanism

Next we asked whether TRPA1 could contribute to the release of inflammatory mediators in bronchoalveolar lavage (BAL) fluid following exposure to CS in mice. BAL taken from wild-type mice (C57BL/6 background [14]) and exposed to CS showed increased levels of a series of mediators, including interleukin-1β (IL-1β), neutrophil chemoattractant chemokine (KC), monocyte chemotactic protein-1 (MCP-1), tissue inhibitor of metalloproteinases-1 (TIMP-1), metalloproteinases-9 (MMP-9), and interleukin (IL) -2, -5, -13. Increases in KC, IL-5, MMP-9, and TIMP-1 by CS were significantly reduced in *Trpa1^−/−^* mouse BAL, indicating a role of TRPA1 in these responses (Figure S4). Information about methods is available online as (Text S1). IL-6, macrophage inflammatory protein-1α (MIP-1α), IL-4, IL-10, and neuropeptides, SP and NKA levels were either below the limits of detection for the assay or were not increased (data not shown).

**Figure 3:**
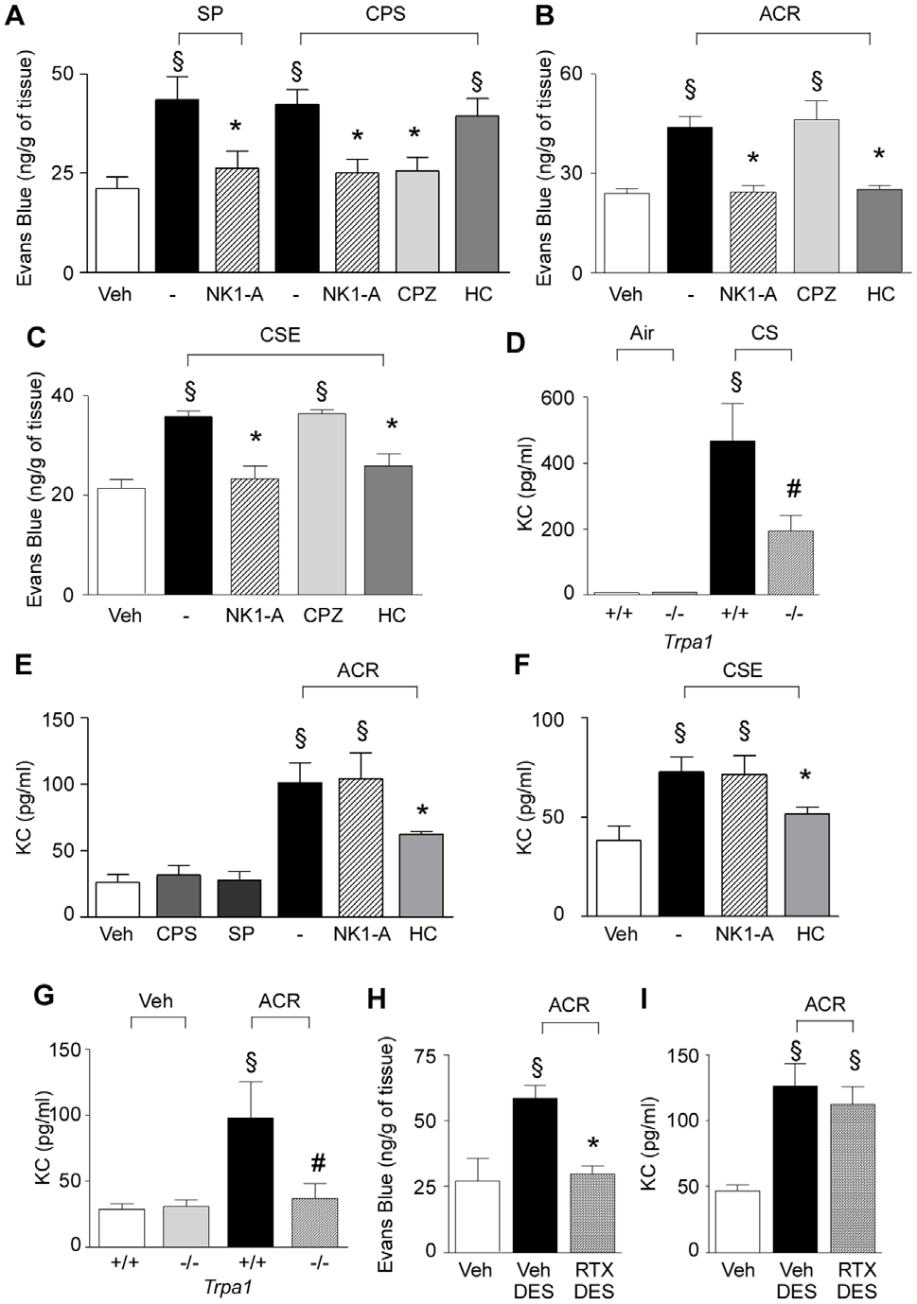
*In vivo* KC release *via* a non-neuronal TRPA1-dependent mechanism. (**A**) Intratracheal instillation (i.t., 30 µl) of substance P (SP, 25 nM), capsaicin (CPS, 100 µM) in C57BL/6 mice increases Evans blue dye extravasation (PPE) in trachea and bronchi an effect prevented by L-733,060 (NK1 receptor antagonist, NK1-A; 2 µmol/kg, i.v.) and capsazepine (CPZ; 30 mg/kg, i.p.) but not by HC-030031 (HC; 300 mg/kg, i.g.). (**B**) L-733,060 and HC, but not CPZ prevent acrolein (ACR, 5 mM, i.t.)- and (**C**) cigarette smoke extract (CSE, 1 OD, i.t.)-evoked increase in PPE in C57BL/6 mouse trachea and bronchi. Each column represents mean ± SEM of at least 5 mice. Veh is the vehicle of the various agonists. ^§^ *P*<0.05 *vs.* Veh; ^*^ *P*<0.05 *vs.* treated group. (**D**) Cigarette smoke (CS)-evoked increase in KC in BAL from *Trpa1^+/+^* is reduced in *Trpa1^−/−^* mice. Each column represents mean ± SEM. ^§^ *P*<0.05 *vs.* air-exposed *Trpa1^+/+^ mice*. ^#^ *P*<0.05 *vs. Trpa1^+/+^* CS group. (**E**) ACR (5 mM, i.t.), but not CPS (100 µM, i.t.) or SP (25 nM, i.t.) increases KC release in BAL. HC, but not L-733,060, reduces the increase in KC release by ACR (**E**) or CSE instillation (1 OD, i.t.) (**F**). (**G**) ACR (5 mM, i.t.)-evoked increase in KC in BAL is practically absent in BAL of *Trpa1^−/−^* mice. Resiniferatoxin-desensitization (RTX-DES) abolished PPE-induced by ACR (5 mM, i.t.) (**H**), while did not modify the ACR-evoked KC release (**I**). Veh-DES is the vehicle of RTX. Each column represents mean ± SEM of at least 5 mice. ^§^ *P*<0.05 vs. Veh or Veh-*Trpa1* ^+/+^; ^*^ *P*<0.05 *vs.* treated group; ^#^ *P*<0.05 *vs.* ACR-*Trpa1* ^+/+^.

CS inhalation has been shown to increase plasma protein extravasation (PPE) in guinea-pig trachea *via* a neurogenic mechanism [4] and TRPA1 receptor activation [20]. In the present study, we confirmed that instillation of acrolein and CSE, as well as capsaicin or SP, into the C57BL/6 mouse tracheal lumen increased PPE, an effect that was prevented by pretreatment with a SP receptor (NK1) antagonist (Figure 3A–C). The effect of acrolein or CSE was selectively abated by HC-030031 and that of capsaicin by the TRPV1 selective antagonist, capsazepine [37] and all responses (including that of SP instillation) by the NK1 receptor antagonist, L-733,060 (Figure 3A–C). Thus, either TRPV1 or TRPA1 stimulants were able to cause neurogenic inflammation in mouse airways, an effect mediated by NK1 receptors.

To investigate whether non-neuronal TRPA1 was involved in the mechanism responsible for inflammatory mediator release by CS, we studied KC release under different circumstances. First, we confirmed using a different colony of *Trpa1^+/+^* and *Trpa1^−/−^* mice (B6129P) and a slightly different protocol (which optimized KC release in BAL) that CS releases KC in BAL from *Trpa1^+/+^* mice, and that this effect was significantly reduced in *Trpa1^−/−^* mice (Figure 3D). Next, we gave capsaicin, SP, acrolein, or CSE to C57BL/6 mice *via* intratracheal instillation, and BAL were collected 24 hours later for KC determination. CSE or acrolein, but not capsaicin or SP, significantly increased KC in BAL (Figure 3E and F), indicating that the effect of CSE or acrolein is not mediated by sensory neuron activation. In addition, pretreatment with HC-030031 significantly reduced KC release, indicating TRPA1 involvement in this phenomenon (Figure 3E and F). Acrolein-induced KC release was completely absent in *Trpa1^−/−^* mice (C57BL/6) (Figure 3G). Noteworthy, in order to exclude the involvement of a neurogenic mechanism in KC release, C57BL/6 mice were pretreated with NK1 receptor antagonist, L-733,060, prior to acrolein or CSE exposure. Indeed, it is known that, upon TRPA1/TRPV1 activation, neuropeptides released from peripheral terminals of primary neurons, including SP, NKA, and CGRP, exert their action through NK1 and NK2 receptors. Here we demonstrated that the pretreatment with NK1 receptor antagonist failed to reduce the increase of KC evoked by either acrolein or CSE (Figure 3E and F), thus excluding a major involvement of neurogenic contribution in the inflammatory response leading to KC accumulation.

Further demonstration of the role of extra-neuronal TRPA1 in KC release was obtained by using resiniferatoxin (RTX), a selective and potent TRPV1 activator [38] which defunctionalizes/destroys TRPV1 expressing afferents, and consequently TRPA1 expressing neurons and neuronal responses to TRPA1 agonists [39], [40]. In C57BL/6 mice treated with a high, desensitizing dose of RTX (50 µg/Kg), acrolein-induced PPE was completely abolished (Figure 3H). In contrast, acrolein-induced increase in KC in BAL fluid (a response mediated by TRPA1 because it is absent in TRPA1-deficient mice) remained unchanged after RTX treatment (Figure 3I).

### Acrolein and CSE exposure releases IL-8 *via* TRPA1 stimulation

**Figure 4:**
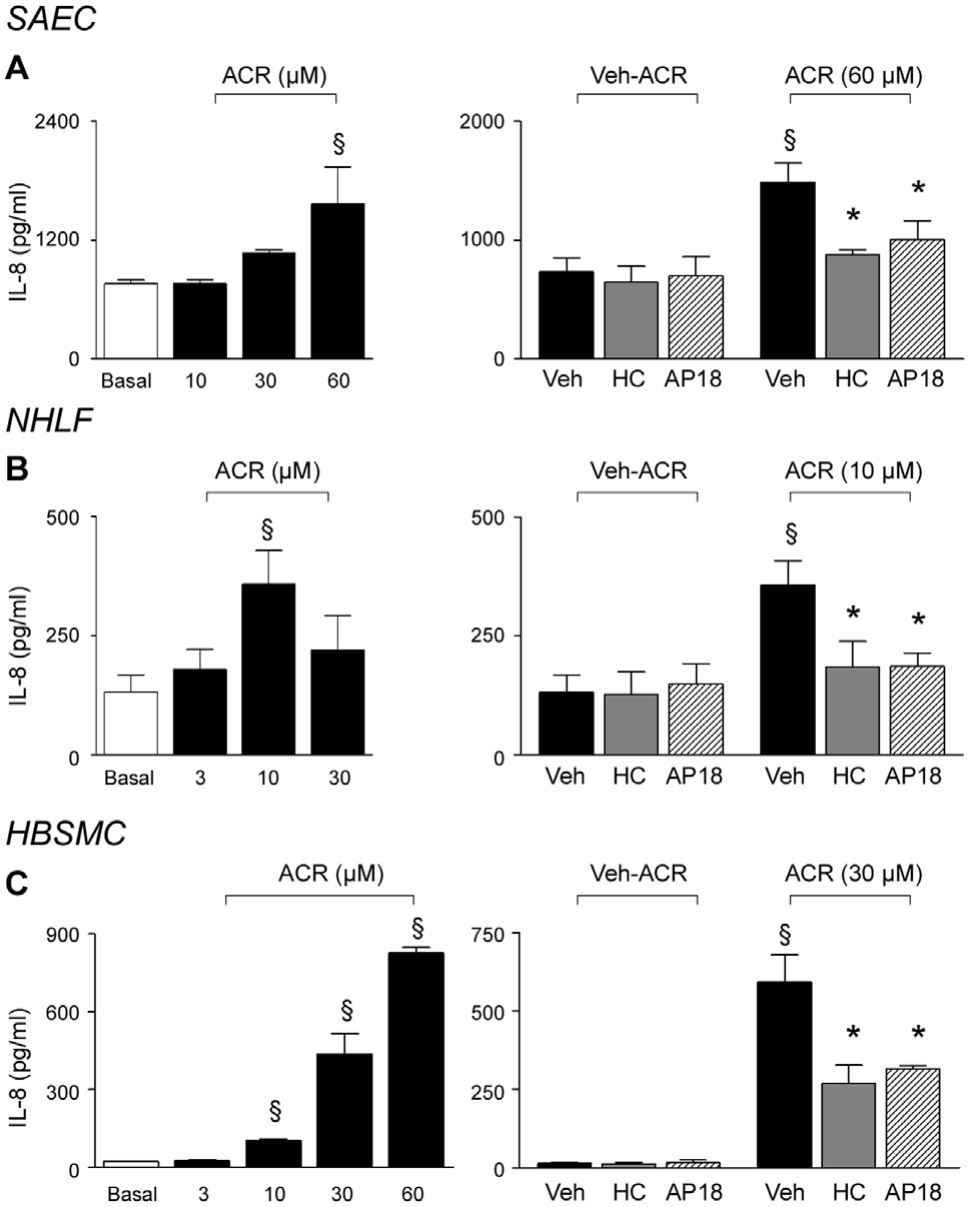
TRPA1 mediates IL-8 release by acrolein from human airway/lung cells in primary culture. Overnight exposure to acrolein (ACR) induces IL-8 release from small airway epithelial cells (SAEC) (**A**), normal human lung fibroblasts (NHLF) (**B**) and human bronchial smooth muscle cells (HBSMC) (**C**) in a concentration–dependent manner. IL-8 release evoked by ACR is reduced by HC-030031 (HC, 30 µM) and AP18 (10 µM). Each column represents mean ± SEM of at least 3 independent experiments. ^§^ *P*<0.05 vs. Basal group or Veh/Veh-ACR; ^*^ *P*<0.05 vs. Veh/ACR.

**Figure 5:**
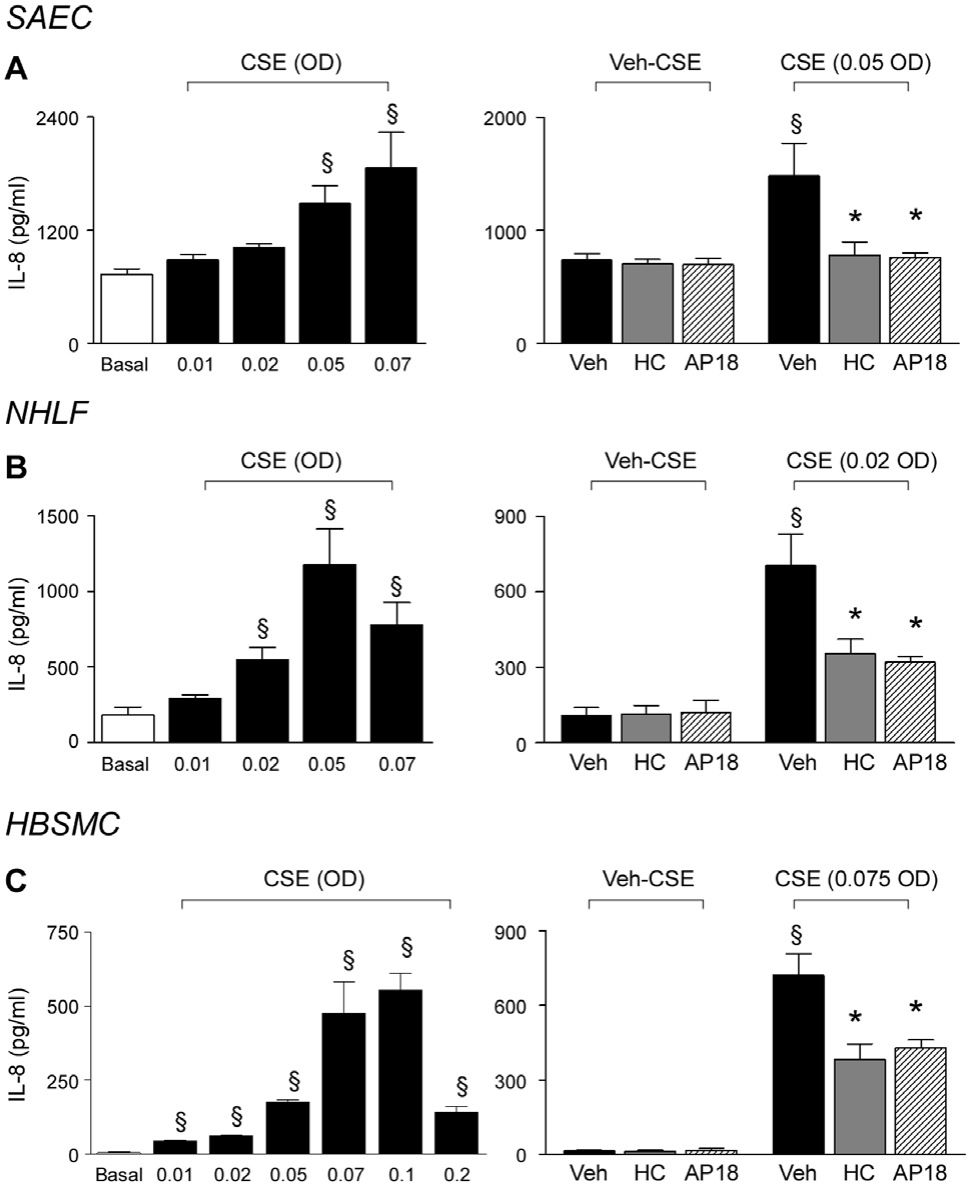
TRPA1 mediates IL-8 release by cigarette smoke extract from human airway/lung cells in primary culture. IL-8 release induced by overnight exposure to increasing concentrations (expressed as optical density, OD) of cigarette smoke extract (CSE) in small airway epithelial cells (SAEC) (**A**), normal human lung fibroblasts (NHLF) (**B**) and human bronchial smooth muscle cells (HBSMC) (**C**). IL-8 release evoked by CSE is reduced by HC-030031 (HC, 30 µM) and AP18 (10 µM). Each column represents mean ± SEM of at least 3 independent experiments. ^§^ *P*<0.05 vs. Basal group or Veh/Veh-CSE; ^*^ *P*<0.05 *vs.* Veh/CSE.

Finally, we explored whether what we found *in vivo* in mice could have a counterpart *in vitro* in human cells. For this purpose we studied whether TRPA1 stimulation could release IL-8 (the human analogue of KC) from human non-neuronal cells of the respiratory tract. We previously demonstrated that acrolein and CSE elicited IL-8 release from either immuno-competent cells, including alveolar macrophages [41], and structural cells, including lung fibroblasts, airway epithelial cells [25] and airway smooth muscle cells [42]. Here, in SAEC, NHLF and HBSMC, we observed that overnight exposure to acrolein evoked a concentration-dependent IL-8 release (Figure 4), a response that was significantly reduced by HC-030031 and AP-18 (Figure 4), suggesting the involvement of the TRPA1 channel in this pathway. Similar results were obtained by using CSE as a stimulus (Figure 5). Overnight exposure to CSE up to 0.1 optical density (OD) did not affect cell viability (Figure S5A–C). CSE induced a concentration (0.01–0.07 OD)- dependent release of IL-8 from SAEC, NHLF and HBSMC, a response that was significantly reduced by pretreatment with TRPA1 antagonists (Figure 5). It should be noted that the action of acrolein and CSE was particularly pronounced in HBSMC, where it was significantly, but not completely, blocked by TRPA1 antagonism (Figure 4C and 5C). The observation that TRPA1 antagonists did not affect IL-8 release evoked by IL-1β or tumor necrosis factor-α (TNF-α), indicated selectivity of the antagonists (Figure S5D–I).

## Discussion

By using functional cellular assays coupled with Real-Time PCR, we demonstrate that TRPA1 is expressed and functional in different human pulmonary non-neuronal cells. In particular, TRPA1 mRNA was detected at various levels of expression in human lung fibroblasts, small airways epithelial cells and smooth muscle cells. In parallel, we performed immunohistochemical and immunofluorescence studies demonstrating the expression of TRPA1 in non-neuronal cells both in mice and human pulmonary tissues. The intense staining found in epithelial and smooth muscle cells in tissues obtained from wild-type mice, and the absence of staining in tissues from TRPA1-deficient mice, strongly supports the proposal that TRPA1 expression localizes to non-neuronal pulmonary cells. The marked staining in trigeminal ganglia (TG; an area enriched with TRPA1 expressing sensory neurons) taken from wild-type mice, compared with the absence of staining in TG taken from TRPA1-deficient mice, conclusively supports the specificity of the antiserum, and strengthens the findings obtained in the mouse respiratory system. The identification of TRPA1 positive staining in human airway tissue which was virtually identical to that found in mice supports a similar extra-neuronal localization of TRPA1 in man.

TRP channels are non-selective cation channels, and their activation usually results in increase in intracellular calcium [9]. The present observation that TRPA1 agonists, cinnamaldehyde, acrolein, and CSE [7], [14], [20], produced calcium responses in airway fibroblasts, epithelial and smooth muscle cells, and selective channel antagonists abated these responses, demonstrated that the TRPA1 channel in these cells is functional. In contrast, the failure of the potent TRPV1 agonist, capsaicin, to elicit any calcium response in any cell type rules out the possibility that cultured cells express functional TRPV1 channels. These functional data are corroborated by failure to detect any TRPV1 staining in human or mouse airway epithelial or smooth muscle cells. The neutrophil chemoattractant, IL-8, may be released from epithelial cell lines following TRPV1 or TRPA1 activation [29], [43], and primary airway fibroblasts and epithelial cells release IL-8 after exposure to acrolein or CSE *in vitro* [25]. Here, we demonstrated that TRPA1 activation in fibroblasts, and epithelial and smooth muscle cells, resulted in the release of IL-8. Present data show that all three TRPA1 activators, cinnamaldehyde, acrolein, and CSE, release IL-8, and TRPA1 antagonists selectively attenuate the response. While cinnamaldehyde has primarily a pharmacological significance, acrolein is one of the major α,β-unsaturated aldehydes present in CS, and CSE contains additional molecules, recently identified as TRPA1 agonists, such as crotonaldehyde [20], acetaldehyde [33], and nicotine [9]. All these compounds may target TRPA1 in sensory nerve terminals to produce an early, neurogenic component of the inflammatory response to CS [20]. However, present findings show that all these agents target TRPA1 also in non-neuronal airway and lung cells to release IL-8.

CS inhalation in rodents results in an early TRPA1-dependent [20] inflammatory response, which in tracheobronchial tissue is mainly represented by PPE [4], a phenomenon entirely mediated by a neurogenic, SP/NKA- and NK1-dependent mechanism [4], [44]. TRPV1 and TRPA1 are co-expressed in a subset of neuropeptide containing primary sensory neurons [10], and their activation is known to promote neurogenic inflammation. Here, we confirm this principle by using the TRPV1 agonist, capsaicin, and TRPA1 agonists, acrolein and CS, which all produced an NK1 receptor-dependent increase in PPE. As expected, intratracheal application of SP, which is released from sensory nerve terminals by both TRPV1 and TRPA1 agonists, increased PPE, *via* NK1 receptor activation. However, when we studied the ability of capsaicin and SP to release KC, the murine analogue of IL-8, a clear dissociation between TRPV1 and the TRPA1 agonists was observed. The two TRPA1 activators, acrolein and CS, were both able to cause a delayed (observed 24 hours after the administration of the stimulus) and robust response. In contrast, capsaicin and SP failed to increase KC in mouse BAL. Thus, in the mouse, *in vivo* TRPA1 agonists increase KC in BAL, whereas capsaicin or SP (the final mediator of neurogenic inflammation) does not. The observation that sensory nerve ablation by RTX [39], [40], while it abolished the neurogenic increase in PPE, failed to affect the KC increase in BAL produced by acrolein, indicates that TRPA1-dependent KC increase does not involve neurogenic mechanisms, and strongly supports the view that TRPV1 and TRPA1 agonists act at different cell types. Capsaicin stimulates TRPV1, whose expression in the airways is selectively confined to sensory nerve terminals, to promote, *via* SP/NKA, neurogenic inflammation. Acrolein and CS, *via* TRPA1, may activate this same neurogenic pathway. However, acrolein and CS also act on non-neuronal TRPA1-expressing cells to promote additional inflammatory responses, such as IL-8/KC release.

These data and conclusions, obtained from *in vitro* experiments in human cells and *in vivo* studies in C57BL/6 mice, were corroborated by genetic studies in TRPA1-deficient mice. The KC release by acrolein was, in fact, abated, and that by CS was markedly reduced in TRPA1-deficient mice. The main aggravant factor in COPD is the irritant effect of CS. A large series of CS constituents have been shown to activate TRPA1 [9], [20], [33], and oxidative stress and its by-products, including 4-hydroxy-2-nonenal, have been proposed to contribute to the mechanism of COPD [45]. Present data suggest that, in rodents, CS exposure first activates an early neurogenic inflammatory response, which is entirely mediated by TRPA1 [4], [20]. However, a second and delayed action of CS, in part mediated by TRPA1, encompasses the release of IL-8/KC. If neurogenic inflammation is not relevant in human airway disease, it is possible that TRPA1 plays a role in COPD because stimulants contained in CS may promote inflammatory mediator release through channel activation in non-neuronal cells. The observation that, in addition to cell lines [29], human epithelial cells in primary culture (fibroblasts, epithelial and smooth muscle cells) express a functional channel and release IL-8 *via* TRPA1 activation suggests that the biological response mediated by non-neuronal TRPA1 observed *in vivo* in mice may be present in humans.

TRPA1 has been proposed as a sensor of byproducts of oxidative and nitrative stress [46], and oxidative stress and its by-products, including 4-hydroxy-2-nonenal, have been suggested to contribute to the mechanism of asthma [47]. Accidental exposure to a number of environmental irritants, many of which are now identified as TRPA1 stimulants [14], [48], [49], [50], [51], has been reported to cause asthma-like symptoms, a condition that has been defined as RADS [52], [53], [54]. The association between acetaminophen use and the increased prevalence of asthma in children [55] has been attributed to the ability of the reactive drug metabolite, N-acetyl-p-benzoquinone imine, to activate TRPA1 in the airways [21]. Present data, proposing that non-neuronal TRPA1, rather than the channel expressed in sensory nerve terminals, contributes to the mechanism of inflammatory airway diseases, offer a novel interpretation for the recent and important finding that ovalbumin-sensitized TRPA1-deficient mice, but not TRPV1-deficent mice, exhibit a much reduced inflammatory phenotype after allergen exposure, with marked reduction in both cytokine release and hyperresponsiveness [22]. If these data demonstrate that TRPV1 is not involved, they suggest a major role for TRPA1. Apart from a recent paper showing that nasally administered SP slightly increases airway hyperresponsiveness in mice [5], to the best of our knowledge, there is no evidence in past or recent literature that pharmacological or genetic interventions on sensory nerves, including sensory nerve desensitization by capsaicin, pharmacological blockade or genetic deletion of NK1/NK2 receptors or TRPV1 channel, abate the major signs of the inflammatory allergic response in mouse airways. In addition, and more importantly, initial [23], [56] and more recent [24] studies show the failure of either selective NK1 receptor antagonists or dual NK1/NK2 tachykinin receptor antagonist to afford protection in asthma. If neurogenic inflammation plays a role in murine models of asthma but no role in human asthma, this does not exclude a TRPA1 contribution to the disease *via* non-neuronal mechanisms. Thus, non-neuronal TRPA1 may be involved in the dramatic inhibition of ovalbumin induced inflammation produced by genetic deletion or pharmacological antagonism of the channel [22].

It should be underlined that in the present experiments the non-neurogenic proinflammatory action of CSE or acrolein, both *in vitro* in human cells and *in vivo* in mice, was not entirely dependent on TRPA1 activation. This is particularly evident in the study of KC release by CS in TRPA1-deficient mice and in the study of IL-8 release by acrolein in bronchial smooth muscle cells. Thus, mechanisms additional to TRPA1 most likely contribute to the early and delayed events associated with the origin and progression of inflammatory airway diseases. In addition, we acknowledge that our analysis is partial, as we have in depth studied only the IL-8/KC release, whereas other mediators, whose release was found to be reduced in TRPA1-deficient mice, were not further investigated. Nevertheless, if this paradigm, based on IL-8/KC, were applicable to other inflammatory mediators and human asthma and COPD, TRPA1 could represent a highly needed novel target, and TRPA1 antagonists might be regarded as novel medicines, for the treatment of inflammatory respiratory diseases by targeting not only the channel expressed in sensory nerve terminals to reduce cough [48], [57], but, more importantly, in resident non-neuronal cells to limit inflammation in airway and lung tissue.

## Materials and Methods

### Ethics Statement

Mice were housed in a temperature- and humidity-controlled vivarium (12 hours dark/light cycle, free access to food and water). Animal experiments were carried out in conformity to the ECC guidelines for animal care procedures and the Italian and Swedish legislation (DL 116/92 and SFS1998:56, respectively) application of the European Communities Council Directive 86/609/EEC. Studies were conducted under the University of Florence researchpermit number 143/2008-B and Chiesi Farmaceutici research permit number 156/2009, approved by the Italian National Committee for animal research and under AstraZeneca R&D research permit reference number 31-11684/08 (Ethical approval number M284/08) and approved by the Ethical Committee for Animal Experiments (Jordbruksverket). Ethical approval for the experiments performed on human tissue was obtained from the Ethics Committee of the University Hospital of the Florence University (Comitato Etico Locale (CEL), Azienda Ospedaliero Universitaria Careggi; approval number 24/09).

### Animals

Male C57BL/6 mice (25 g) (Harlan Laboratories), wild-type (*Trpa1^+/+^*) or TRPA1-deficient mice (*Trpa1^−/−^*) generated by heterozygous mice on a C57BL/6 background kindly donated by Prof. D. Julius (UCSF, CA) [14], were used. Some i*n vivo* experiments, as indicated B6129P in the results section, were performed in mice derived from crosses of *Trpa1^−/−^* (B6;129P-*Trpa1tm1Kykw*/J) and wild-type (B6129PF2/J) mice (Jackson Laboratories), generated by heterozygous mice on a C57BL/6 background. All experiments with TRPA1-deficient and wild-type littermates were performed blinded to the genotype. Animals were sacrificed with a high dose of i.p. sodium pentobarbital (200 mg/kg).

### Cell Culture

Human small airway epithelial cells (SAEC), normal human lung fibroblasts (NHLF) and human bronchial smooth muscle cells (HBSMC) were purchased from Lonza. Human alveolar type II epithelium-like adherent cell line (A549) and human lung embryonic fibroblast (IMR90) were purchased from American Type Culture Collection (Manassas, VA). SAEC and NHLF were cultured respectively in SAGM and FBM medium (Lonza) according to the manufacturer's instructions. IMR90 and HBSMC were cultured in DMEM supplemented with 10% FBS, 2 mM glutamine, 100 U penicillin and 100 µg/ml streptomycin (Sigma-Aldrich), and A549 were cultured in RPMI supplemented with 10% FBS, 2 mM glutamine, 100 U penicillin, 100 µg/ml streptomycin and 1 mM HEPES (Sigma-Aldrich). All cells were cultured in an atmosphere of 95% air and 5% CO_2_ at 37°C.

### Production of cigarette smoke extract (CSE)

CSE was generated as previously reported [41]. Briefly, aqueous CSE was obtained from the combustion of four cigarettes (Marlboro Red, 12 mg of tar, 0.9 mg of nicotine each) bubbled through 50 ml of buffer and subsequently filtered through a 0.2-µm pore filter (Millipore). To ensure reproducibility among different CSE batches, the absorbance (optical density; OD) measured at 320 nm was used as a measure of the “strength” of the extract. Dilutions were made with buffer to obtain the desired absorbance. The CSE was freshly prepared on the day of the experiment and used immediately after preparation.

### Real-Time PCR

For Real-Time PCR studies, cells were seeded in complete culture medium in 96-well and grown to ∼80–90% confluence. Adherent cells were rinsed with cold PBS and cell lysates, and reverse transcriptase reactions were performed using the TaqMan Gene Expression Cells-to-Ct Kit (Applied Biosystems). Briefly, cells were lysed in Cell Lysis solution containing DNAse I for 5 min, followed by two-min incubation with the stop solution. Cell lysates were immediately used for RT reactions; 50 µl reverse transcription reactions were performed using 10 µl of each cell lysate, according to the manufacturer's instructions. Two sets of primers-probes were designed using the Primer Express Software version 3.0 (Applied Biosystems). The chosen reporter fluorophores for TaqMan MGB probes were VIC for the endogenous reference β-actin gene (ACTB) and 6-carboxyfluorescein (FAM) for TRPA1 gene. The two sets of primers-probes were as follows: set 1, ACTB-FW (forward) 5′- GGCGGCACCACCATGTAC-3′, ACTB-RE (reverse) 5′-CAGGGCAGTGATCTCCTTCTG-3′, ACTB probe 5′-VIC-TGGCATTGCCGACAGG-3′; and set 2, TRPA1-FW (forward) 5′- GGCAGTTGGCGACATTGC-3′, TRPA1-RE (reverse) 5′-CTGATCCACTTTGCGTAGAAACC-3′ and TRPA1 probe 5′-FAM-CATCATTGAAGAGGATAGCTA-3′. The chosen primers and probes were subjected to Basic Local Alignment Search Tool (BLAST) database searches to find any sequence similarities. Real-Time quantitative PCR was performed using StepOnePlus™ Real-Time PCR System (Applied Biosystems, Foster City, CA). All samples were run in triplicate in a final volume of 25 µl containing 12.5 µl of 2× TaqMan Gene Expression PCR Master Mix, 300 nM of each primer, 250 nM of each probe and 4 µl of RT reaction, according to the manufacturer's instructions (Applied Biosystems). Amplification conditions were: 50°C for 2 min and 95°C for 10 min, followed by 40 cycles of 95°C for 30 s and 60°C for 1 min. Relative expression of TRPA1 mRNA was calculated using the 2-^Δ^(^Δ^CT) comparative method, with each gene normalized against the internal endogenous reference β-actin gene for the same sample.

### Immunohistochemistry

Sections of 4 µm thickness were cut from formalin-fixed, paraffin-embedded samples of human normal lung tissue obtained from patients who were undergoing surgery for lung cancer. All specimens seemed macroscopically normal, without signs of tumor or inflammation. Sections were processed with rabbit polyclonal TRPA1 antibody (1∶250 overnight 4°C, Novus Biologicals) [29]. Negative control has been obtained by substituting the primary antibody with normal serum. In another set of experiments, *Trpa1^+/+^* or *Trpa1^−/−^* mice were perfused *via* the right ventricle with 20 ml of PBS, and lung and trigeminal ganglia were removed in 10% formalin, and after embedding in paraffin, 4 µm sections were immunohistochemically stained with rabbit polyclonal TRPA1 antibody (1∶500, 2 hours 37°C, AVIVA System Biology) which recognizes the middle region of the protein, (residues 540–589) or with rabbit polyclonal TRPV1 antibody (1∶200, overnight 4°C, Alomone Labs). Specificity of staining was determined by preadsorption with immunizing peptide (1∶250, overnight 4°C, AVIVA Biosystem). Serial sections of mouse and human lung tissues were processed with anti-cytokeratin (1∶250, overnight 22°C, Abcam), anti-α-smooth muscle actin (1∶200, overnight 22°C, Abcam) and anti-TRPV1 (1∶200, overnight 4°C, Alomone Labs) antibodies. Immunostaining was performed according to standard procedures. Briefly, the slides were dewaxed in Bio-Clear (Bio-Optica) and hydrated with grade ethanol concentrations until distilled water. Antigen retrieval was routinely performed by immersing the slides in thermostat bath containing 10 mM citrate buffer (pH 6.0) for 15 min at 97°C followed by cooling for 20 min at room temperature. Endogenous peroxidase activity was blocked by treating the sections with 3% hydrogen peroxide in distilled water for 10 min. After blocking with normal horse serum (UltraVision), sections were incubated with the respective antibodies. Bound antibodies were visualized using aminoethylcarbazol and 3.3′diaminobenzidine (LabVision) as chromogens. Nuclei were counterstained with Mayer's haematoxylin. Negative controls were performed by substituting the primary antibody with a nonimmune serum. Details for immunofluorescence method are available online as (Text S1).

### Calcium Imaging

For calcium fluorescence measurements, cells were incubated with 5 µM Fura-2AM ester (Alexis Biochemicals) for 40 minutes at 37°C. Intracellular calcium levels were measured and recorded with a dynamic image analysis system (Laboratory Automation 2.0; RC Software). Fluorescence was measured during excitation at 340 and 380 nm, and after correction for the individual background fluorescence signals, the ratio of the fluorescence at both excitation wavelengths (Ratio_340/380_) was monitored. Experiments were performed using buffer solution containing (in mM): 150 NaCl, 6 KCl, 1 MgCl2, 1.5 CaCl2, 10 glucose, 10 HEPES at pH 7.4. Cells were stimulated with cinnamaldehyde (0.1–3000 µM), acrolein (0.1–3000 µM), CSE (0.001–0.5 OD), capsaicin (1 µM) and the activating peptide (SLIGKV-NH2) of the human PAR-2 receptor (hPAR-2 AP; 100 µM), or their respective vehicles (0.3% DMSO,aqueous buffer or 1% ethanol). HC-030031 and AP18 vehicles (used in all the *in vitro* experiments) were 3% DMSO and 1% DMSO, respectively. Results are expressed as the increase of Ratio_340/380_ over the baseline normalized to the maximum effect induced by ionomycin (5 µM) added at the end of the experiment.

### Desensitization to resiniferatoxin

To defunctionalize/desensitize TRPV1 expressing primary sensory neurons, resiniferatoxin (RTX) was administered to C57BL/6 mice, as previously described [39], [40]. Briefly, mice were treated subcutaneously (s.c.) with a single injection of RTX (50 µg/kg) or its vehicle (10% ethanol and 10% tween 80 in isotonic saline) into the scruff of the neck under a light ether anesthesia to avoid unnecessary pain. Experiments were carried out 7 days after RTX treatment when responses to both TRPV1 and TRPA1 agonists in the treated mice were abolished [39], [40].

### Plasma Protein Extravasation

Anesthetized (sodium pentobarbital, 50 mg/kg i.p.) C57BL/6 mice received Evans blue dye (30 mg/kg i.v.), 1 min before intratracheal (i.t) instillation (30 µl) of acrolein (5 mM), capsaicin (100 µM), SP (25 nM), CSE (1 OD) or their vehicles (isotonic saline, 1% ethanol in isotonic saline and isotonic saline). Drugs were given to mice through the intratracheal route after exposing the trachea and by inserting a 30-gauge needle just below the inferior laryngeal rim. The tachykinin NK1 receptor antagonist, L-733,060 (2 µmol/kg i.v.; Tocris) or its vehicle were administered 15 min before the stimulus. The TRPV1 selective antagonist, capsazepine, (30 mg/kg i.p.) or its vehicle (2.5% DMSO in isotonic saline) and intragastric (i.g.) TRPA1 selective antagonist, HC-030031 (300 mg/kg i.g.) or its vehicle were administered 30 and 60 min before the stimulus, respectively. In another set of experiments ACR (5 mM, i.t.), or its vehicle, has been administered in C57BL/6 mice desensitized to RTX (50 µg/kg; s.c.). Animals were euthanized 15 min after treatment and transcardially perfused with 0.9% isotonic saline solution. The extravasated dye was extracted from mouse trachea and bronchi by overnight incubation in formamide and assayed by spectrophotometry at 620 nm, as reported previously [20].

### Bronchoalveolar Lavage

*Trpa1^+/+^* or *Trpa1^−/−^* B6129P mice (5 per group) were exposed acutely to the cigarette smoke (CS) produced with commercial cigarettes (Marlboro Red, 12 mg of tar, 0.9 mg of nicotine each), by using a “nose-only” exposure unit. The smoke produced by cigarette burning was introduced into the exposure chamber (inner volume = 11.61 l) with the airflow generated by a mechanical ventilator (7025 Rodent Ventilator, Ugo Basile) at a rate of 250 ml/min [58]. A second mechanical ventilator was used to provide room air for dilution (1∶8) of the smoke stream. Mice were exposed to the smoke of 5 cigarettes per exposure period, twice daily, for 3 consecutive days, and sacrificed 24 hours later. Genotype-, age-, strain- and sex-matched control animals were exposed to air only in the same manner for the same duration of time. Anesthetized C57BL/6, and *Trpa1^+/+^* or *Trpa1^−/−^* (C57BL/6) mice were intratracheally administered with (30 µl) acrolein (5 mM), capsaicin (100 µM), SP (25 nM) and CSE (1 OD) or their vehicles (isotonic saline, 1% ethanol in isotonic saline and isotonic saline) and sacrificed 24 hours later. The NK1 receptor antagonist, L-733,060 (2 µmol/kg i.v.; Tocris) or its vehicle (isotonic saline) were administered 15 min before the stimulus, and i.g. HC-030031 (300 mg/kg) or its vehicle (0.5% CMC in isotonic saline 0.9%) were administered 1 hour before the stimulus. In another set of experiments ACR (5 mM, i.t.), or its vehicle, has been administered in C57BL/6 mice desensitized to RTX (50 µg/kg; s.c.). Animals were sacrificed 24 hours after the last exposure/treatment with an overdose of anesthetic followed by exsanguination, and the lungs were lavaged using a cannula inserted into the trachea and instilling 1 ml of Hank's Buffer added with HEPES 10 mM and EDTA 10 mM three times. Routine recovery of BAL did not significantly differ between animals with ∼80% of instilled volume recovered. BAL were centrifuged at 200× g at 4°C for 10 min, and the cell-free supernatants were stored at −80°C for cytokine determination. Frozen cell-free BAL was analyzed for keratinocyte chemoattractant (CXCL-1/KC, the murine analogue of IL-8) release by using a commercial ELISA-assay (Invitrogen).

### IL-8 release and ELISA assay

For IL-8 ELISA assays, HBSMC, SAEC, and NHLF were seeded in complete culture medium in 48-well plates, grown to ∼80–90% confluence, and incubated overnight in serum-free medium before treatments. All the treatments were performed in serum free medium. Cells were pretreated with the selective TRPA1 antagonists, HC-030031 and AP18, or their vehicles, for 30–60 min before treatment with freshly prepared acrolein, CSE, TNF-α and IL-1β for 18 hours at 37°C with 5% CO2. Subsequently, supernatants were collected and stored at −80°C for ELISA assay. Human IL-8 was measured using a paired antibody quantitative ELISA kit (Invitrogen) (detection limit: 5 pg/ml). The assays were performed according to the manufacturer's instructions. Vitality assay method (MTT reduction assay) is available online as (Text S1).

### Statistical Analysis

Analyses were performed using GraphPad Prism 5 statistical software. All data were expressed as mean ± SEM or Confidence Interval (CI). Agonist potency was expressed as EC_50_, that is, the molar concentration of agonist producing 50% of the measured effect. Statistical significance was determined by using one- or two-way ANOVA, followed by Bonferroni's post hoc analysis for comparison of multiple groups and the unpaired 2-tailed Student's t-test between 2 groups. P<0.05 was considered significant.

## Supporting Information

Figure S1 **Immunoistochemical and immunofluorescent staining of TRPA1 protein.** (**A**) Immunoistochemical and immunofluorescent staining for TRPA1 protein in sections of trigeminal ganglia obtained from *Trpa1^+/+^* and *Trpa1^−/−^* mice. Double labeling fluorescence with cytokeratin or α-smooth muscle actin (α-SMA) (red) and TRPA1 (green) antibodies in human (B) and *Trpa1^+/+^* mice (**C**) airways/lung tissues. Scale bar 100 µm for immunohistochemistry and 50 µm for immunofluorescence.
(TIF)

Figure S2 **Immunoistochemistry of TRPV1 in mouse and human airways and in trigeminal ganglia obtained from mice.** TRPV1 staining is absent in epithelial or smooth muscle cell layers in mouse and human airways/lung tissues (**A** and **B**). (**C**) Immunoistochemical staining for TRPV1 protein in sections of mouse trigeminal ganglia. Scale bar 100 µm.
(TIF)

Figure S3 **Functional TRPA1 receptors are expressed in human airway/lung cells.** Typical traces and concentration-dependent intracellular calcium response induced by selective TRPA1 agonists, cinnamaldehyde (CNM, typical traces and black circles) and acrolein (ACR, grey circles), and by cigarette smoke extract (CSE, black triangles) in human type II alveolar epithelial cell line (A549) (**A**) and human fetal lung fibroblasts (IMR90) (**B**). The calcium response evoked by CNM, ACR or CSE both in A549 (**A**) and IMR90 (**B**) is abolished by selective TRPA1 antagonists, HC-030031 (HC, 10 µM) or AP18 (10 µM). Calcium response elicited by stimulation with PAR-2 receptor activating peptide (SLIGKV-NH_2_) (PAR-2 AP, 100 µM) is not affected by TRPA1 antagonists, indicating selectivity. Veh is a combination of vehicles of HC and AP18. Values represent mean ± SEM of n>25 cells. ^§^ *P*<0.05 *vs.* Veh.
(TIF)

Figure S4 **Inflammatory mediators analysis of BAL fluid taken from** ***Trpa1^+/+^*** **and** ***Trpa1*** **^−/−^ mice exposed to cigarette smoke (CS) for 5 consecutive days.** CS exposure increases significantly KC, IL-5, MMP9, TIMP-1, IL-1β, IL-2, MCP-1, IL-13 levels in *Trpa1* ^+/+^ mice BAL. Increases in KC, IL-5, MMP-9 and TIMP-1 evoked by CS exposure are significantly reduced in BAL of *Trpa1* ^−/−^ mice. Each column represents mean ± SEM of at least 5 mice *per* group. ^*^ *P*<0.05 *vs.* air-exposed *Trpa1^+/+^ mice*. ^#^ *P*<0.05 *vs. Trpa1^+/+^* CS group.
(TIF)

Figure S5 **IL-8 release from cultured cells of the human respiratory tract by IL-1β or TNF-α is not mediated by TRPA1.** IL-8 release induced by overnight exposure to IL-1β or to TNF-α in small airway epithelial cells (SAEC) (**A, D**), normal human lung fibroblasts (NHLF) (**B, E**) and human bronchial smooth muscle cells (HBSMC) (**C, F**) is not affected by TRPA1 antagonists, HC-030031 (HC, 30 µM) and AP18 (10 µM). Each column represents the mean ± SEM of at least 3 independent experiments. ^§^ *P*<0.05 vs. Basal group or Veh/Veh-ACR; ^*^ *P*<0.05 vs. Veh/ACR. Effect of cigarette smoke extract (CSE) exposure on cell viability evaluated by using the [3-(4,5-dimethylthiazol- 2-yl)-2,5-diphenyltetrazolium bromide] (MTT) test in small airway epithelial cells (SAEC) (**G**), normal human lung fibroblasts (NHLF) (**H**) and human bronchial smooth muscle cells (HBSMC) (**I**). Each column represents mean ± SEM of at least 3 independent experiments ^§^ *P*<0.05 *vs.* Basal group.
(TIF)

Text S1 **Methods.**
(DOCX)
