# Supplementary figures and images for: Transient Receptor Potential Ankyrin 1 Channel Localized to Non-Neuronal Airway Cells Promotes Non-Neurogenic Inflammation
Source: PLoS One. 2012 Aug 14;7(8):e42454. doi: 10.1371/journal.pone.0042454 (PMC3419223; doi:10.1371/journal.pone.0042454)

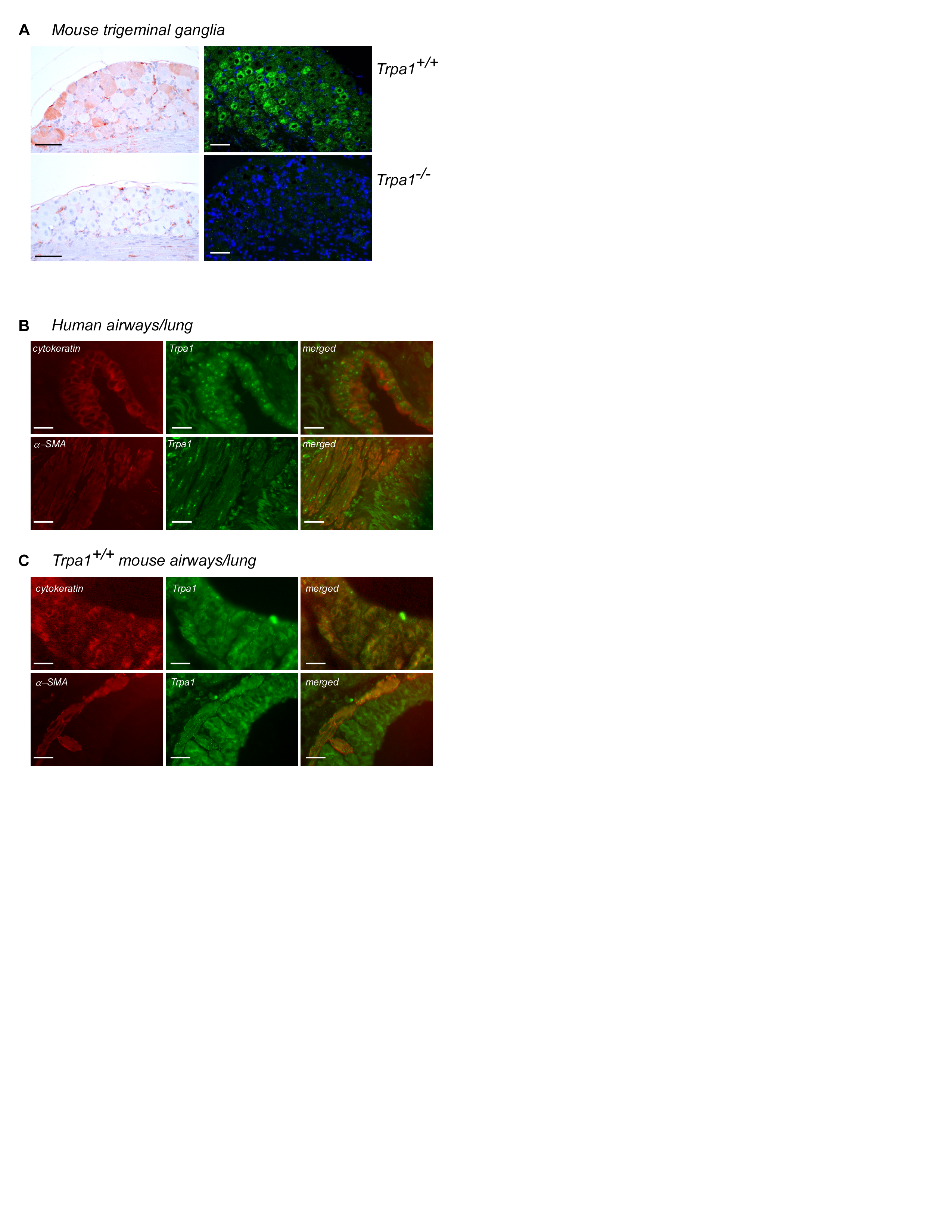

Supplement: Figure S1 — Immunoistochemical and immunofluorescent staining of TRPA1 protein. (A) Immunoistochemical and immunofluorescent staining for TRPA1 protein in sections of trigeminal ganglia obtained from Trpa1+/+ and Trpa1−/− mice. Double labeling fluorescence with cytokeratin or α-smooth muscle actin (α-SMA) (red) and TRPA1 (green) antibodies in human (B) and Trpa1+/+ mice (C) airways/lung tissues. Scale bar 100 µm for immunohistochemistry and 50 µm for immunofluorescence. (TIF) [file pone.0042454.s001.tif]

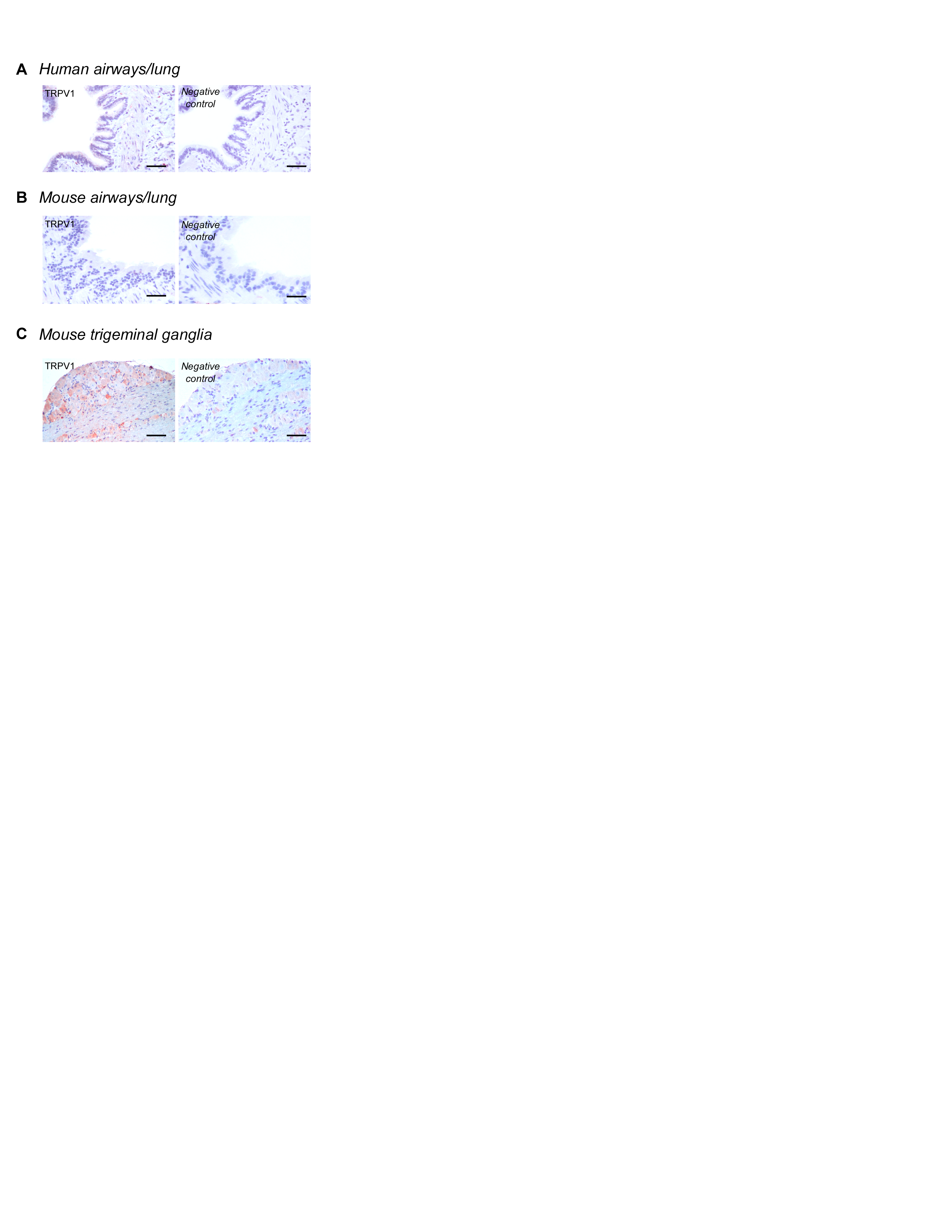

Supplement: Figure S2 — Immunoistochemistry of TRPV1 in mouse and human airways and in trigeminal ganglia obtained from mice. TRPV1 staining is absent in epithelial or smooth muscle cell layers in mouse and human airways/lung tissues (A and B). (C) Immunoistochemical staining for TRPV1 protein in sections of mouse trigeminal ganglia. Scale bar 100 µm. (TIF) [file pone.0042454.s002.tif]

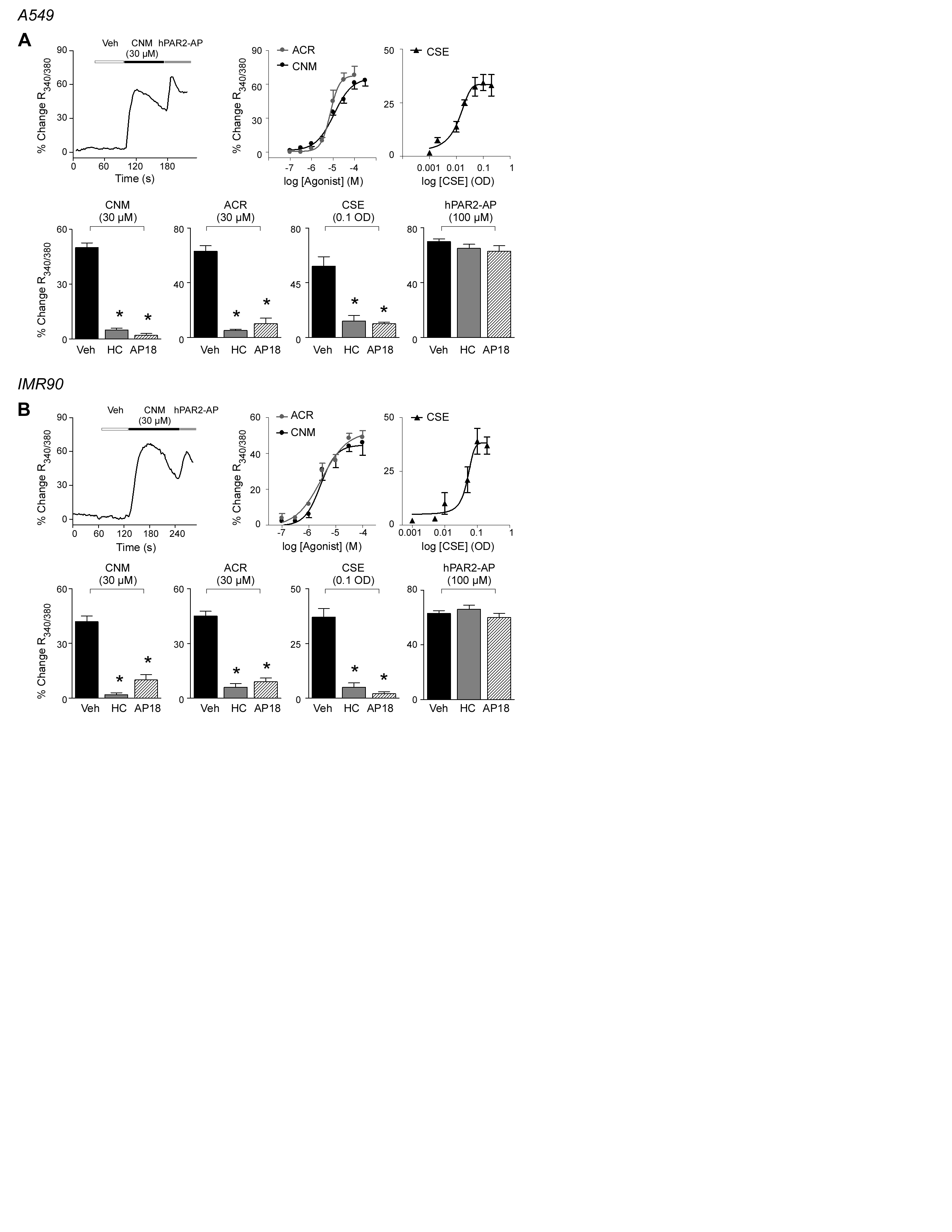

Supplement: Figure S3 — Functional TRPA1 receptors are expressed in human airway/lung cells. Typical traces and concentration-dependent intracellular calcium response induced by selective TRPA1 agonists, cinnamaldehyde (CNM, typical traces and black circles) and acrolein (ACR, grey circles), and by cigarette smoke extract (CSE, black triangles) in human type II alveolar epithelial cell line (A549) (A) and human fetal lung fibroblasts (IMR90) (B). The calcium response evoked by CNM, ACR or CSE both in A549 (A) and IMR90 (B) is abolished by selective TRPA1 antagonists, HC-030031 (HC, 10 µM) or AP18 (10 µM). Calcium response elicited by stimulation with PAR-2 receptor activating peptide (SLIGKV-NH2) (PAR-2 AP, 100 µM) is not affected by TRPA1 antagonists, indicating selectivity. Veh is a combination of vehicles of HC and AP18. Values represent mean ± SEM of n>25 cells. § P<0.05 vs. Veh. (TIF) [file pone.0042454.s003.tif]

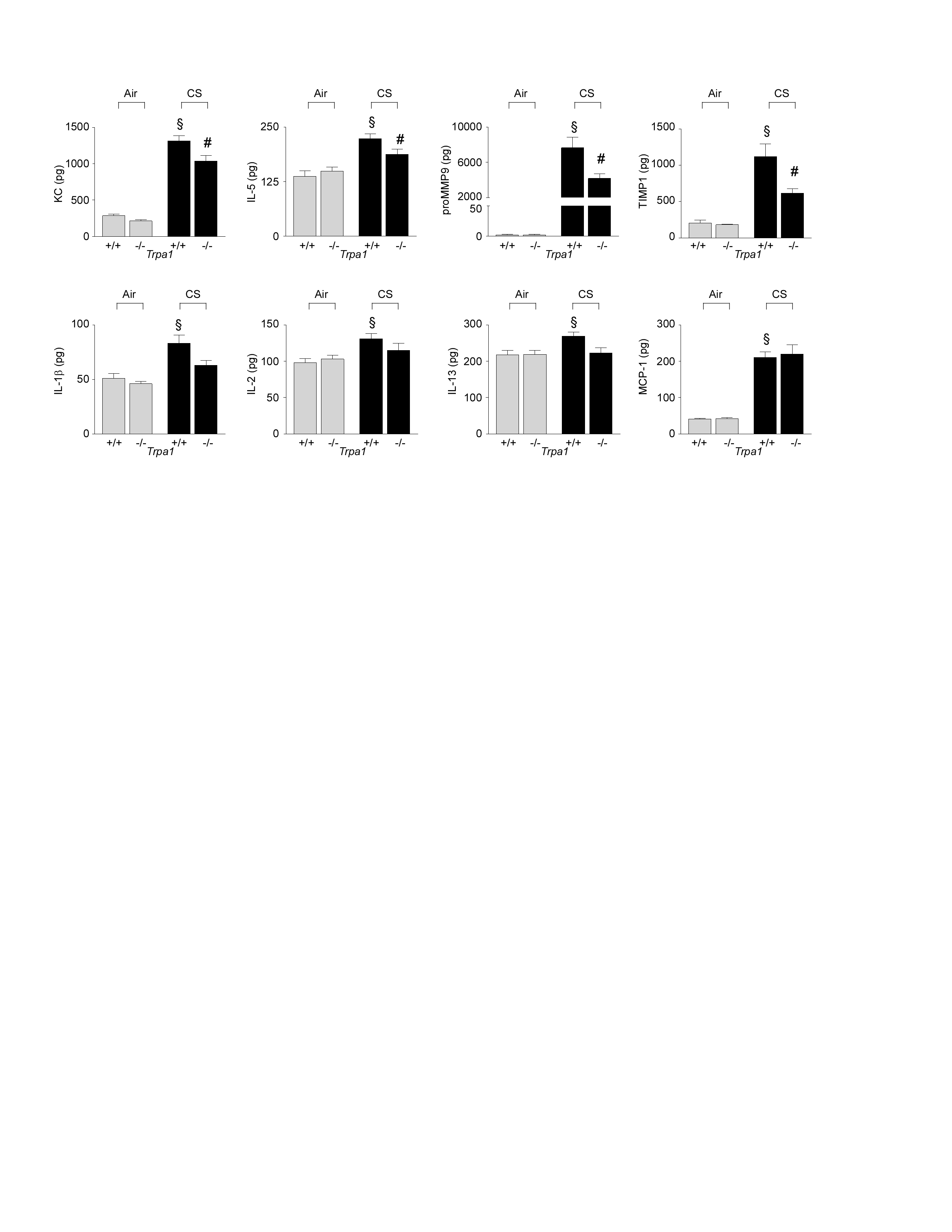

Supplement: Figure S4 — Inflammatory mediators analysis of BAL fluid taken from Trpa1+/+ and Trpa1 −/− mice exposed to cigarette smoke (CS) for 5 consecutive days. CS exposure increases significantly KC, IL-5, MMP9, TIMP-1, IL-1β, IL-2, MCP-1, IL-13 levels in Trpa1 +/+ mice BAL. Increases in KC, IL-5, MMP-9 and TIMP-1 evoked by CS exposure are significantly reduced in BAL of Trpa1 −/− mice. Each column represents mean ± SEM of at least 5 mice per group. * P<0.05 vs. air-exposed Trpa1+/+ mice. # P<0.05 vs. Trpa1+/+ CS group. (TIF) [file pone.0042454.s004.tif]

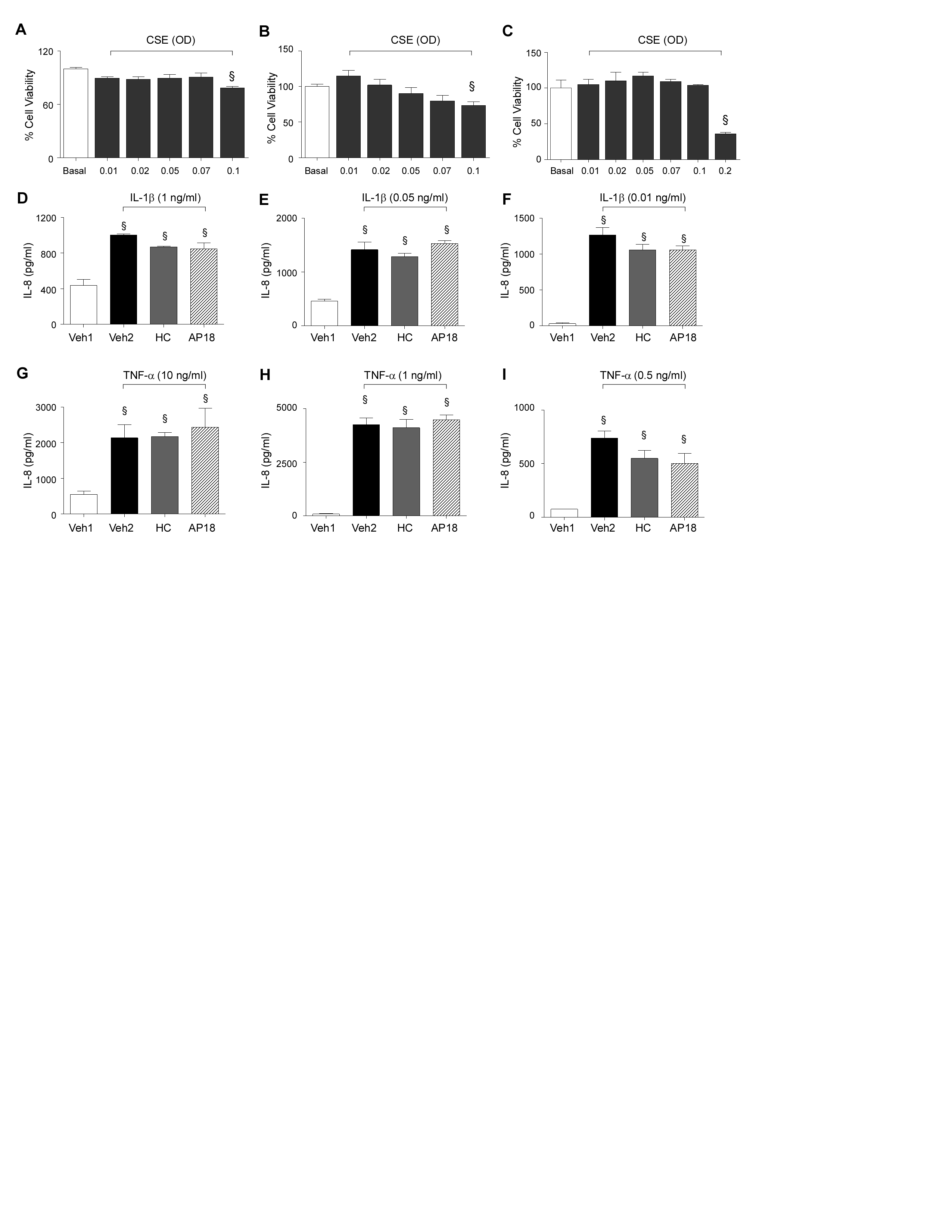

Supplement: Figure S5 — IL-8 release from cultured cells of the human respiratory tract by IL-1β or TNF-α is not mediated by TRPA1. IL-8 release induced by overnight exposure to IL-1β or to TNF-α in small airway epithelial cells (SAEC) (A, D), normal human lung fibroblasts (NHLF) (B, E) and human bronchial smooth muscle cells (HBSMC) (C, F) is not affected by TRPA1 antagonists, HC-030031 (HC, 30 µM) and AP18 (10 µM). Each column represents the mean ± SEM of at least 3 independent experiments. § P<0.05 vs. Basal group or Veh/Veh-ACR; * P<0.05 vs. Veh/ACR. Effect of cigarette smoke extract (CSE) exposure on cell viability evaluated by using the [3-(4,5-dimethylthiazol- 2-yl)-2,5-diphenyltetrazolium bromide] (MTT) test in small airway epithelial cells (SAEC) (G), normal human lung fibroblasts (NHLF) (H) and human bronchial smooth muscle cells (HBSMC) (I). Each column represents mean ± SEM of at least 3 independent experiments § P<0.05 vs. Basal group. (TIF) [file pone.0042454.s005.tif]
