# Supplementary material for: Transient Receptor Potential Ankyrin 1 Channel Localized to Non-Neuronal Airway Cells Promotes Non-Neurogenic Inflammation
Source: PLoS One. 2012 Aug 14;7(8):e42454. doi: 10.1371/journal.pone.0042454 (PMC3419223; doi:10.1371/journal.pone.0042454)
Supplement: Text S1 — Methods. (DOCX) [file pone.0042454.s006.docx]

**Supporting Information**

**Text S1 Methods**

***Immunofluoscence*.**

For fluorescence staining, mouse trigeminal ganglia and human and mouse lung sections of 4 µm thickness were dewaxed in Bio-Clear (Bio-Optica) and hydrated with grade ethanol concentrations until distilled water, then, placed in blocking solution (PBS, pH 7.4 and 0.3% Triton X-100, 2 g/l BSA and 5% NGS), for 30 min at room temperature. Sections of mouse trigeminal ganglia and lung were processed with a rabbit polyclonal TRPA1 antibody (1:500, AVIVA System Biology) diluted in fresh blocking solution and applied 2 hours at 37°C. Sections of human lung were processed with a rabbit polyclonal TRPA1 antibody (1:250 overnight 4 °C, Novus Biologicals). Serial sections of mouse and human lung tissues were also incubated with the mouse monoclonal anti-cytokeratin (1:250, overnight 22 °C, Abcam) and mouse monoclonal anti-α-smooth muscle actin (1:200, overnight 22 °C, Abcam). Sections were then incubated for 2 hours in the dark with the appropriate fluorescent secondary antibody (polyclonal anti-goat Alexa Fluor 488 green conjugated and monoclonal anti-mouse Alexa Fluor 594 red conjugated; Invitrogen) diluted 1:200 in blocking solution. Sections were finally coverslipped using Vectashield water-based mounting medium with DAPI (Vector Laboratories). The analysis of negative controls (nonimmune serum) was simultaneously performed in order to exclude the presence of non-specific immunofluorescent staining, cross-immunostaining, or fluorescence bleed-through.

***Vitality assay by the tetrazolium salt method (MTT reduction assay).***

Cytotoxicity of CSE and of any compound tested on SAEC and NHLF was assessed by using the 3-(4,5-dimethylthiazol-2-yl)- 2,5-diphenyltetrazolium bromide (MTT) viability test. MTT was added to the culture medium at a final concentration of 0.5 mg/ml and incubated at 37 °C for 1 h. The reaction product of MTT was extracted in DMSO, and the OD was spectrophotometrically measured at 570 nm with DMSO as a blank. Viability was expressed as percentage of the values (corresponding to 100%) of untreated cells.

***Cigarette smoke exposure and BAL.***

The *Trpa1^+/+^* or *Trpa1^-/-^* C57BL/6 mice were positioned in a randomized way in the exposure box at every smoke exposure occasion and exposed to cigarette smoke for 50 min twice daily day 1-4. The flow was 1.5 L/min. Mice were sacrificed on day 5 (24 hours after the last smoke exposure). Animals were lavaged directly after termination according to the standard protocol by cannulation of the trachea and administration of 2 ml PBS. Weight of harvested BAL was measured. BAL was kept on ice until centrifugation step (Rotanta 46R, 1200 rpm, 10 min, 4 °C). Supernatant was removed and saved at -80 °C for mediator analysis, such as IL-1β, KC, IL-6, TIMP-1, MCP-1, MMP-9, MIP-1α, as well as IL-2,-4,-5,-10,-13, and neuropeptides SP and NKA in BAL were analyzed through a paired antibody quantitative ELISA kit (R&D System). The assays were performed according to the manufacturer’s instructions. The significance of differences between groups has been calculated by using Student t-test with one-tailed distribution and two-sample unequal variance.
